# Shenfu Injection Protects Brain Injury in Rats with Cardiac Arrest through Nogo/NgR Pathway

**DOI:** 10.1155/2022/4588999

**Published:** 2022-12-26

**Authors:** Haixia Deng, Zhanhong Tang, Peng Tuo, Ruihua Wu, Si Jia, Xuan Zhao, Deqing Huang, Yuguang Gao, Zhou Lan

**Affiliations:** ^1^Department of Surgical Intensive Care Unit, The First Affiliated Hospital of Guangxi Medical University, Nanning 530021, China; ^2^Department of Emergency, The First Affiliated Hospital of Guangxi University of Chinese Medicine, Nanning 530022, China; ^3^Guangxi University of Chinese Medicine, Nanning 530200, China

## Abstract

The effect of Shenfu injection on brain injury after cardiac arrest (CA) and cardiopulmonary resuscitation (CPR) along with the underlying mechanism of axonal regeneration was explored. CA/CPR model in rats was established for subsequent experiments. A total of 160 rats were randomly divided into sham group, model group, conventional western medicine (CWM) group, Shenfu group, and antagonist group (*n* = 32 per group). After 3 hours, 24 hours, 3 days, and 7 days of drug administration, the modified Neurological Severity Score tests were performed. The ultrastructure of the brain and hippocampus was observed by electron microscopy. Real-time quantitative polymerase chain reaction (PCR), western blotting, and immunohistochemistry were used to detect Nogo receptor (NgR) expression in the hippocampus and cerebral cortex, and Nogo–NgR expression in CA/CPR model. Neurological deficits in the model group were severe at 3 hours, 24 hours, 3 days, and 7 days after the recovery of natural circulation, whereas the neurological deficits in CWM, antagonist, and Shenfu group were relatively mild. The ultrastructure of neuronal cells in Shenfu group had relatively complete cell membranes and more vesicles than those in the model group. The results of PCR and western blotting showed lower messenger ribonucleic acid and protein expression of NgR in Shenfu group than the model group and CWM group. Immunohistochemical examination indicated a reduction of Nogo–NgR expression in Shenfu group and antagonist group. Our results suggested that Shenfu injection reduced brain injury by attenuating Nogo–NgR signaling pathway and promoting axonal regeneration.

## 1. Introduction

Cardiac arrest (CA) is one of the leading causes of mortality worldwide, with approximately 375,000–700,000 cases each year in Europe [1] and an estimated 500,000 in the United States [2]. Cardiopulmonary resuscitation (CPR) is an emergency measure for CA patients, which enhances their chance of survival, thereby reducing the impact of heart attack on brain function. The recovery rate of cardiopulmonary cerebral resuscitation (CPCR) is low. The efficiency of CPCR is known to be closely related to the prognosis and quality of patients' life. Therefore, it is necessary to improve the success rate of CPR to avoid brain damage caused by hypoxia [3].

Cerebral ischemia–reperfusion injury is often present during CA/CPR process. Neurons in the central nervous system usually do not regenerate after damage due to the presence of proteins in mature oligodendrocytes [4]. Nogo protein, a myelin-derived axon growth inhibitor, can bind to the Nogo receptor (NgR) complex distributed on the surface of axon growth cones to inhibit axon regeneration. The inhibitory effects of the membrane protein Nogo and its receptor NgR on axonal growth, axonal regeneration, and plasticity in the central nervous system, such as the mechanism in spinal cord injury repair and optic nerve axon has been extensively studied [5–7].

Shenfu injection is an extract of red aconite, which has been widely used in the treatment of many heart-related diseases, such as cardiogenic shock, coronary heart disease, acute myocardial infarction, and myocarditis [8]. ZF wang et al. [9] have confirmed that Shenfu injection has a good protective effect on cerebral ischemia–reperfusion injury in rats. It is believed that Shenfu injection can alleviate ischemic injury in heart and brain; however, the specific mechanism of Shenfu has not yet been fully explained.

We speculated that the effect of Shenfu injection on axonal regeneration may be associated with the Nogo/NgR signaling pathway. In this study, the CA/CPR rat models were established to investigate the protective effect of Shenfu on brain injury and to explore whether the mechanism of axonal regeneration is involved in Nogo/NgR signaling pathways.

## 2. Materials and Methods

### 2.1. Animals

A total of 160 adult male Sprague–Dawley rats (weighing 300–350 g) were purchased from Shanghai Xipuer Bikai Experimental Animal Co., Ltd., Shanghai, China. After one week of acclimatization, all animals were housed in the specific-pathogen-free rooms under a standard light environment (12 hours light–dark cycle) at the temperature of 23 ± 2°C. Rats were given free access to normal standard chow diet and water. This study was conducted in strict accordance with The International Guidelines for Care and Use of Laboratory Animals. The experimental protocols were approved by the Ethics Committee of The First Affiliated Hospital of Guangxi University of Traditional Chinese Medicine, Nanning, China. All procedures were performed under sufficient anesthesia and analgesia to minimize the suffering of animals.

### 2.2. Grouping

Rats were randomly divided into 5 groups (*n* = 32 per group); (1) sham group: exposed body but not operated on plus the administration of 1 mL normal saline intraperitoneally; (2) model group: rats with CA received CPR and injected intraperitoneally with 1 mL normal saline after return of spontaneous circulation (ROSC); (3) conventional western medicine (CWM) group: the rats with CA received CPR and CWM treatment of epinephrine, dopamine, and mild hypothermia after ROSC; (4) Shenfu group: the CA rats received Shenfu injection (2.1 mL/kg, Z51020664, China Resources Sanjiu Yaan Pharmaceutical Co., Ltd., Shenzhen, China) immediately after ROSC; (5) antagonist group: rats with CA received CPR and NogoA antagonists (10 mg/kg) immediately after ROSC. Each group was then categorized into 4 subgroups (*n* = 8 per subgroup) according to the different time period of drug administration as follows: 3 hours, 24 hours, 3 days, and 7 days.

### 2.3. CA/CPR Rat Models

All rats were fasted overnight and had only free access to water before the operation. The rats were anesthetized through intraperitoneal injection ketamine (50 mg/kg) and xylazine (10 mg/kg) and placed supine on the operation table. The left femoral artery and vein were isolated for cannulation. A catheter was inserted into the left femoral vein and connected to a microinfusion pump (Suzhou Xunfei Scientific Instrument Co., Ltd., Suzhou, China) to inject normal saline (2 mL/hour) continuously. An F24 catheter (Beijing Zhongyuan Jianbang Medical Equipment Co., Ltd., Beijing, China) was inserted from the left femoral artery to the thoracic aorta and connected to the animal electrocardiogram monitor (SurgiVet V3404, Youcheng Company of Hong Kong, Shenzhen, China) through a transducer (ZH0144, China Huaibei Zhenghua Company, Huaibei, China) to check arterial blood pressure and heart rate. After completion of the above processes, endotracheal intubation was carried out, and rats were connected to a volume-controlled ventilator (TKR-200C, Beijing Yatai Kelong Company, Beijing, China) at the tidal volume of 15 mL/kg, FiO_2_ of 0.21, and ventilation rate of 12–20 breaths/minutes. An in-line infrared capnograph (Beijing Zhongheng Rixin Technology Co., Ltd., Beijing, China) was placed in the airway for end-tidal PCO_2_ monitoring. The ventilation rate and the tidal volume were controlled to maintain normocapnia (35–45 mmHg) of rats. Arterial blood gases were also analyzed by ABL90 Blood Gas Analyzer (Radiometer Medical Equipment (Shanghai) Co., Ltd., Shanghai, China) to confirm baseline ventilation.

The CA rat models were successfully established using asphyxiation combined with intravenous potassium chloride solution [10]. Rats with disappeared spontaneous cardiac rhythm waveforms and less than 30 mmHg of mean arterial pressure were considered CA rats.

After 5 minutes of CA on rats, CPR was performed with chest compressions (160–200 beats/minutes) by an experienced CPR technician in the laboratory. Meanwhile, the rats were ventilated by a small animal ventilator with a respiratory rate of 80 beats/minutes, a tidal volume of 2.5 mL, and 100% pure oxygen. After that, epinephrine (0.01 mg/kg) and atropine (0.01 mg/kg) were injected into the femoral vein. If necessary, the rats were taken an additional dose of epinephrine every 3 minutes until ROSC was achieved. ROSC was defined as a regular cardiac rhythm with the mean aortic pressure greater than 60 mmHg for at least 10 minutes [11].

### 2.4. Specimen Collection

After administration drugs at different time points, rats were sacrificed by cervical dislocation after being treated with ketamine (50 mg/kg) and xylazine (10 mg/kg). Cerebral tissue samples of rats were collected for real-time quantitative polymerase chain reaction (PCR; RT-qPCR), western blotting, and immunohistochemistry, whereas the hippocampus were taken for electron microscopy examination and RT-qPCR. All the brain samples were frozen and stored in liquid nitrogen at −80°C.

### 2.5. Neurobehavioral Tests

The modified Neurological Severity Score (mNSS) test was applied to evaluate the neurofunctional behavior of rats at the corresponding time points of drug administration [12, 13], which included motor, sensory, reflex, and balance assessments with the highest score of 18. The scoring of mNSS test was classified as 13–18: severe injury; 7–12: moderate injury; and 1–6: mild injury.

### 2.6. Electron Microscopy

A portion of hippocampus tissues was taken and cut into 1 *μ*m, followed by fixation with 2.5% glutaraldehyde and 1% osmic acid. The tissues were then dehydrated through an ethyl alcohol–acetone gradient and embedded in Epon812. Tissues were sectioned as 60 nm by an Ultra-thin microtome (Leica EM UC6, Leica Microsystem GmbH, Wetzlar, Germany) and stained by uranyl acetate and lead citrate. After that, the ultrastructure changes of hippocampus tissues were observed under a transmission electron microscope (Hitachi, Tokyo, Japan).

### 2.7. Real-Time Quantitative PCR

Total RNA was extracted by TRIzol (American Invitrogen Company, CA, USA) following the manufacturer's instructions. The extracted RNAs were transformed into complementary DNA (cDNA) through a Reverse Transcription Reagent Kit (American GeneCopoeia Company, Guangzhou China). After that, cDNA was amplified by the Real-Time PCR detection system for observation. (American Applied Biosystems, MA, USA) with the following primer sequences: NgR (forward: 5′-TTCCGTCCCTTCCAGACCAA-3′; reverse: 5′-CATTGCCTGGTGGAGTGTCA-3′), Nogo (forward: 5′-TTCCCACGTTTGTCAGTGCT-3′; reverse: 5′-ATCTGCACCTGATGCCGTTC-3′), and glyceraldehyde-3-phosphate dehydrogenase (GAPDH) (forward: 5′-TGGAATCCTGTGGCATCCATGAAAC-3′; reverse: 5′-TAAAACGCAGCTCAGTAACAGTCCG-3′). GAPDH was used as loading control.

### 2.8. Western Blotting

Brain samples were rinsed with phosphate buffered saline (PBS) and lysed by radio-immunoprecipitation assay lysis buffer containing protease inhibitors and phosphatase inhibitors. The solutions were centrifuged to collect the supernatant, followed by extracting protein according to the manufacturer's protocol. Proteins were then separated by 10% sodium dodecyl sulfate-polyacrylamide gel electrophoresis and transferred to polyvinylidene fluoride membranes. The membranes were blocked in 5% non-fat milk in Tris-buffered saline/Tween-20 (50 mM Tris, pH 7.5, 500 mM sodium chloride, and 0.05% Tween-20) for 1 hour and incubated with 1 : 200 diluted Rabbit Anti-Nogo (Abcam, Cambridge, UK) and anti-NgR polyclonal antibody (H-120, Santa Cruz Biotechnology, Santa Cruz, CA, USA) overnight at 4°C. Subsequently, the membranes were placed at room temperature for 1 hour with goat anti-rabbit secondary antibody (Wuhan Bode Bioengineering Co., Ltd., Wuhan, China) conjugated to horseradish peroxidase. An enhanced Chemiluminescence Detection System (Tanon, Shanghai, China) was carried out to detect the protein bands by a Geliance 600 Imaging System (PerkinElmer Instruments (Shanghai) Co., Ltd., Shanghai, China) with a cooled 12-bit camera. Proteins were finally quantified by densitometry.

### 2.9. Immunohistochemistry

NgR monoclonal antibodies were purchased from Santa Cruz Biotechnology. All procedures were following the manufacturer guidelines. Cells from brain with brown granular in cytoplasm or nucleus were considered as Nogo or NgR positive cells under light microscopy, whereas 0.01 mmol/L PBS instead of primary antibody was added into negative control. No immunological reactions were observed in the negative control. Non-overlapping areas of the cortex and hippocampus from 4 serial slices in each rat were randomly selected under a 40-fold microscope, and the parts stained brown in each field of view were observed.

### 2.10. Statistical Analysis

Statistical analysis was performed with SPSS 20.0 software. Continuous variables were presented as the means ± standard deviation (SD). Comparisons between groups were done with one-way analysis of variance (one-way ANOVA) with Bonferroni post-hoc tests. *P* < 0.05 was considered statistically significant.

## 3. Results

### 3.1. Neurological Function Scores

**Figure 1:**
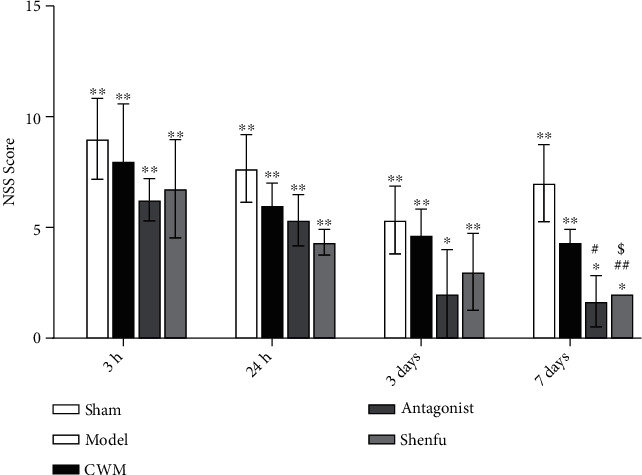
The neurological function scores (mNSS) in each group at 3 hours, 24 hours, 3 days, and 7 days. *n* = 8 in each subgroup at different time. Compared with the Sham group, ∗*P* < 0.05 and ∗∗*P* < 0.01; compared with the model, ^#^*P* < 0.05 and ^##^*P* < 0.001; compared with the conventional western medicine, ^$^*P* < 0.05.

The higher the mNSS, the severer the neurological dysfunction in rats. The results of sham group showed no neurological deficits at 3 hours, 24 hours, 3 days, and 7 days after ROSC, whereas the degree of neurological impairment in model group was much severer than CWM group, antagonist group, and Shenfu group (Figure 1). In addition, the mNSS of the model group was significantly different from the antagonist group and Shenfu group at day 7 (*P* < 0.01). Compared with CWM group, the mNSS of antagonist group and Shenfu group at different time periods were lower, with no significant difference. There were also no significant differences in mNSS between the antagonist group and the Shenfu group.

### 3.2. Ultrastructure of Hippocampus Tissues

**Figure 2:**
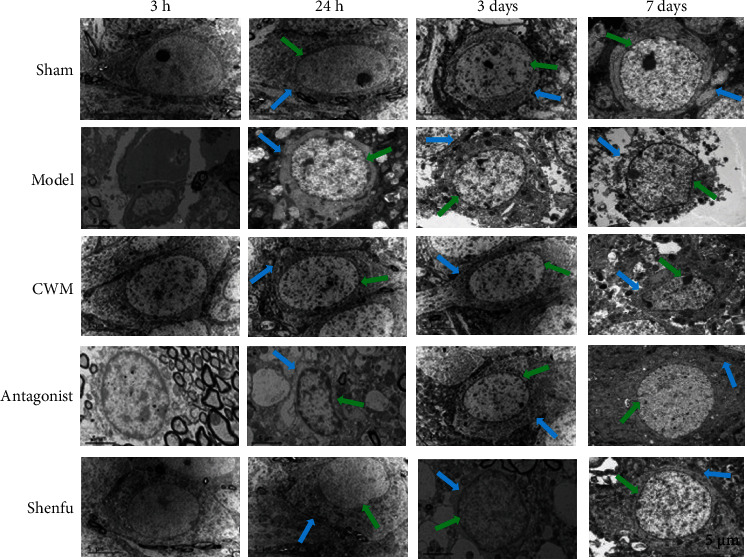
Ultrastructural changes of hippocampal tissue under electron microscopy. Green arrows mean cell membrane of neuron. Blue arrows mean nucleoli.

As shown in Figure 2, compared with the sham group, the cell ultrastructure of hippocampus tissue in model group showed ruptured cell membrane of neuron cells, irregular nucleoli, blurred mitochondrial crest, and reduced number of synaptic vesicles. Yet, the structures of hippocampus tissues in CWM group, antagonist group, and Shenfu group had relatively complete cell membranes and an increased number of vesicles than model group, indicating that the CWM, Shenfu, and antagonist all had protective effect on the ultrastructure of neurons.

### 3.3. The mRNA Expression of NgR

**Figure 3:**
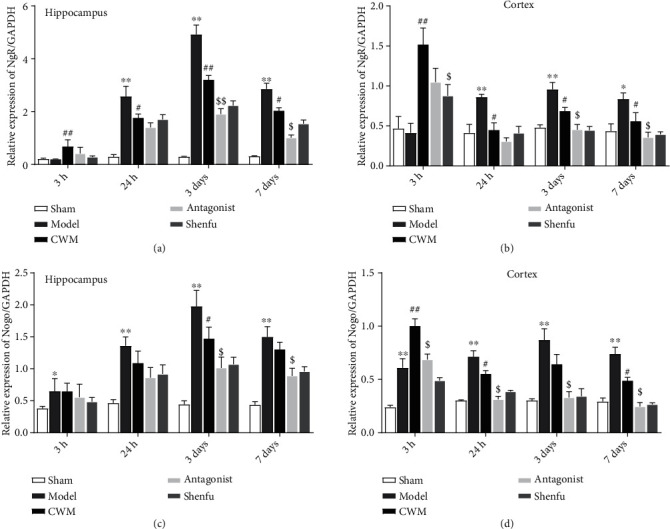
The relative messenger ribonucleic acid expression of Nogo and Nogo receptor in hippocampal tissues (a) and (c) and cerebral cortex tissues (b) and (d) at 3 hours, 24 hours, 3 days, and 7 days after return of spontaneous circulation (ROSC). Compared with the sham group, ∗*P* < 0.05 and ∗∗*P* < 0.01; compared with the model, ^#^*P* < 0.05 and ^##^*P* < 0.01; compared with the conventional western medicine, ^$^*P* < 0.05 and ^$$^*P* < 0.001. *n* = 3 in each subgroup at different times.

The messenger ribonucleic acid (mRNA) expressions of Nogo and NgR in the hippocampus and cortex at 3 hours, 24 hours, 3 days, and 7 days after ROSC were demonstrated in Figure 3. From 3 hours to 3 days, the mRNA expression of Nogo and NgR in the hippocampus and cortex was significantly increased time-dependently compared than that in the sham group. At 3 hours, the mRNA expression of NgR in CWM, antagonist, and Shenfu group was also increased obviously compared with the sham group (Figures 3(a) and 3(b)). NgR expression in CWM, antagonist, and Shenfu group was significantly decreased compared with model group at 24 hours, 3 days, and 7 days. More critically, the antagonist and Shenfu group showed more effects on downregulation NgR expression. It was similar to NgR, Nogo expression in the hippocampus and cortex was increased in model group compared with sham group, which was suppressed by CWM, antagonist, and Shenfu at 24 hours, 3 days, and 7 days (Figures 3(c) and 3(d)). It was observed that both Nogo and NgR in hippocampus were highest at 3 days in model group and antagonist as well as Shenfu treatment attenuated NgR and Nogo expression in hippocampus much more significantly. Therefore, the hippocampus from animals at 3 days was further assayed in the present study.

### 3.4. The Protein Expression of Nogo–NgR

**Figure 4:**
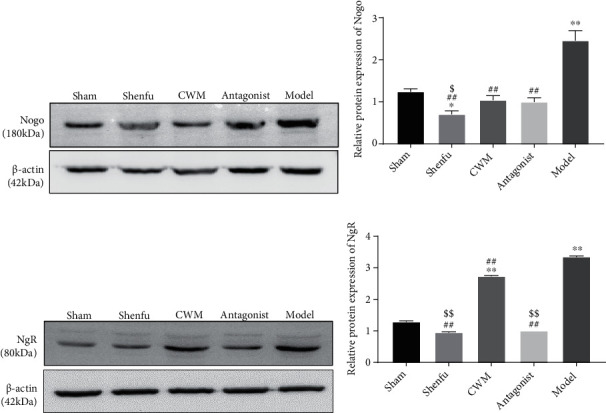
Western blotting detection of Nogo and NgR protein expression in each group at 3d after return of spontaneous circulation (ROSC). (a) Western blots of Nogo in hippocampal tissues of each group. (b) Nogo protein expression analyzed in hippocampal tissues of each group. (c) Western blots of NgR in hippocampal tissues of each group. (d) NgR protein expression analyzed in hippocampal tissues of each group. Compared with the Sham group, ∗*P* < 0.05, ∗∗*P* < 0.01; compared with the model, ^##^*P* < 0.01; compared with the CWM, ^$^*P* < 0.05, ^$$^*P* < 0.001. *n* = 3 in each subgroup at different time.

The protein expression of Nogo and NgR in hippocampal tissues at 3 days was assayed by western blotting group (Figure 4(a, c)). Compared with other groups, Nogo and NgR expressions in the model group were increased significantly (*P* < 0.001), whereas Nogo and NgR expression in Shenfu group were obviously lower than that in CWM group (*P* < 0.001; Figure 4(b, d)), suggesting that Shenfu injection had a great inhibitory effect on Nogo and NgR expression than CWM.

### 3.5. The Expression of NgR

**Figure 5:**
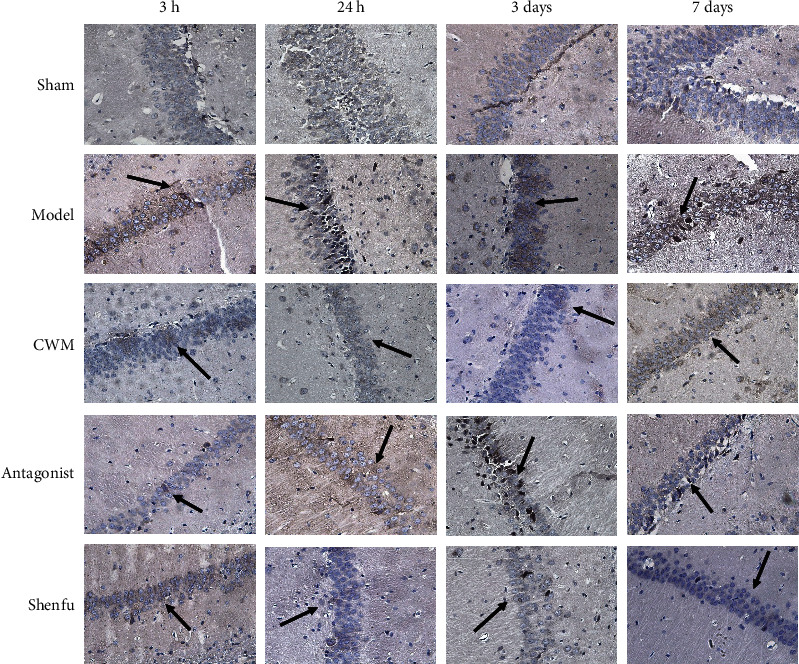
Immunohistochemical detection of Nogo receptor positive cells indicated by black arrows in hippocampal tissues of each group.

Immunohistochemical staining was used to observe the expression of NgR in brain of each group at 3 hours, 24 hours, 3 days, and 7 days after CPR (Figure 5). At different time periods, the number of NgR positive cells in the antagonist group was the lowest. In the Shenfu group, the expression of NgR positive cells reached a peak at 24 hours, followed by a decrease at 3 days. The overall changes of positive cells in Shenfu group were similar to the antagonist group. After 3 days, the number of NgR positive cells in Shenfu group was less than the model group and CWM group. The above results suggested the administration of Shenfu injection and antagonist could decrease the expression of NgR positive cells.

## 4. Discussion

An early work has demonstrated the protective effects of Shenfu injection on cerebral ischemia–reperfusion injury in rats [9]; however, the specific mechanism of Shenfu injection has not been fully clarified. Hence, this study aimed to evaluate the effects of Shenfu injection on brain injury after CA/CPR. The results showed that Shenfu injection inhibited NgR expression in the hippocampus and cerebral cortex of the rat, as well as the expression level of Nogo–NgR, suggesting that Shenfu injection may serve as a protector in the brain to promote axonal regeneration by blocking Nogo–NgR signaling pathway.

Shenfu injection contains the main active ingredients ginsenosides and aconitine. In clinical practice, Shenfu injection is not only used to treat coronary heart disease [14] but also to prevent ischemia–reperfusion related injury. Previous studies have reported that Shenfu injection can effectively prevent acute kidney injury by improving energy metabolism and inhibiting cell apoptosis [15]. Furthermore, Shenfu injection also has beneficial effects on cerebral ischemia–reperfusion injury and craniocerebral injury. The administration of Shenfu injection can prevent neuron cell apoptosis in neonates with hypoxic–ischemic brain damage [16]. As we know, the higher the mNSS of the group, the severer the neurological dysfunction in rats. In this study, the lowest mNSS of Shefu group was observed, which means Shenfu injection significantly improved the recovery of nerve function after cerebral injury caused by CA. This finding is consistent with those previous studies. Besides, a study conducted by Qin et al. [17] has found that Shenfu injection may alleviate brain damage by increasing brain metabolism. Shenfu injection has impact on the expression of apoptosis-regulating genes (*Bcl-2* and *bax*) and reduces apoptosis of nerve cells after hypoxia and ischemia in newborn mice [17]. Similar results were observed in this study that the cells from the hippocampus had relatively complete cell membranes and an increased number of vesicles after Shenfu injection treatment. Therefore, Shenfu injection played a protective role in brain injury by reducing hippocampus damage and cortical structure in rats.

Numerous studies have shown that Nogo/NgR pathway plays a significant part in plasticity, axonal regeneration, and axonal repair. Wei-Ping Xiao et al. [18] have clarified that electroacupuncture could promote axonal regeneration by inhibiting Nogo/NgR and Rho/ROCK signaling pathways in rats with spinal cord injury. Shuhei Ito et al. [19] have also suggested that lateral olfactory tract usher substance, an NgR antagonist, has neuroprotective and regenerative effects on the injured spinal cord. In our experiment, we observed that the expression of NgR in the hippocampus and cerebral cortex of rats was down-regulated after the administration of Shenfu injection to the cerebral injury. Furthermore, results of mRNA expression of NgR and Nogo in hippocampal tissues and cerebral cortex tissues showed that both of NgR and Nogo in hippocampal tissues of model rats tend to be highest at 3 days after CPR. Moreover, NgR and Nogo mRNA expression in hippocampal was more significant compared with that in cerebral cortex tissues. Therefore, the protein expression of NgR and Nogo in hippocampal tissues at 3 days after CPR was measured along with morphological changes in the hippocampus in our study. Meanwhile, in the antagonist group, NgR expression in the hippocampus and cerebral cortex of the rats was also down-regulated after the injection of Nogo-66 receptor antagonist, indicating the expression of NgR was correlated with the neurological function and cell apoptosis of rats. Thereby, the results may suggest that Shenfu injection can promote the regeneration of axons by attenuating Nogo/NgR signaling pathway in rats with brain injury.

It has been reported in previous study that Shenfu injection might show anti-inflammatory or anti-oxidative effects on myocardial ischemia–reperfusion injury, sepsis, and acute lung injury in rats [20–23]. Nogo–NgR was associated with axonal regeneration, and inhibition of Nogo–NgR could attenuate nerve injury in previous studies [24–26]. Results of our study indicated that Shenfu injection may suppress CA-induced Nogo–NgR up-expression in the hippocampus of rats and improve neurological function, which suggested that Shenfu injection might protect the brain in rats with CA and be helpful for axonal regeneration through Nogo/NgR pathway. As Nogo–NgR is involved in the different downstream responses, we would explore profound molecular mechanisms of Shenfu effects in our further study, which may be inadequacy of our present study.

Taken together, this study was the first one to report the effect of Shenfu injection on rats with CA and discussed the role of Nogo/NgR signaling pathway in promoting axonal regeneration. Our study provided a theoretical basis for the application of Shenfu injection in brain injury after CA; however, the efficacy of Shenfu injection still needs to be further studied.

## 5. Conclusions

In conclusion, Shenfu injection effectively protects the brain from CA by alleviating the damage to hippocampal tissues and cerebral cortex tissues and regulating Nogo/NgR signaling pathway for axonal regeneration.

## Data Availability

Data supporting this research article are available from the corresponding author or first author on reasonable request.

## Abbreviations

CA: Cardiac arrest
CPCR: Cardiopulmonary cerebral resuscitation
CPR: Cardiopulmonary resuscitation
CWM: Conventional western medicine
mNSS: Modified neurological severity score
NgR: Nogo receptor
ROSC: Return of spontaneous circulation.
